# Genome-Wide Identification of PR10 Family Members in Durum Wheat: Expression Profile and In Vitro Analyses of TdPR10.1 in Response to Various Stress Conditions

**DOI:** 10.3390/plants13223128

**Published:** 2024-11-07

**Authors:** Emna Khanfir, Ikram Zribi, Hanen Dhouib, Mouna Ghorbel, Karama Hamdi, Olfa Jrad, Inès Yacoubi, Faiçal Brini

**Affiliations:** 1 Biotechnology and Plant Improvement Laboratory, Centre of Biotechnology of Sfax, University of Sfax, P.O. Box 1177, Sfax 3018, Tunisia; emna.khanfir@cbs.rnrt.tn (E.K.); ikram.zribi@cbs.rnrt.tn (I.Z.); karama.hamdi@cbs.rnrt.tn (K.H.); olfa.jrad@cbs.rnrt.tn (O.J.); ines.bouchrityaccoubi@cbs.rnrt.tn (I.Y.); 2 Biopesticides Laboratory, Centre of Biotechnology of Sfax, University of Sfax, P.O. Box 1177, Sfax 3018, Tunisia; hanen.dhouib@cbs.rnrt.tn; 3 Department of Biology, College of Sciences, University of Hail, P.O. Box 2440, Ha’il City 81451, Saudi Arabia; m.ghorbel@uoh.edu.sa

**Keywords:** abiotic and biotic stress, antifungal activity, durum wheat, genome-wide analysis, LDH activity, PR10 family, phytohormones, RNase activity

## Abstract

The functional characterization of PR10 proteins has been extensively studied in many plant species. However, little is known about the role of TdPR10 in the response of durum wheat (*Triticum durum* Desf.) to stress. In this study, we identified members of the *T. durum* PR10 family, which are divided into three major subfamilies based on phylogenetic analyses. The analysis revealed that tandem duplication was the primary driver of the expansion of the *T. durum PR10* gene family. Additionally, gene structure and motif analyses showed that *PR10* family genes were relatively conserved during evolution. We also identified several cis-regulatory elements in the *TdPR10* promoter regions related not only to abiotic and biotic stress but also to phytohormonal responses. In response to abiotic stresses and phytohormones, several *TdPR10* genes were highly expressed in the leaves and roots of durum wheat. Moreover, TdPR10.1 family members improve RNase activity, increase LDH protective activity under abiotic stress conditions, and ensure resistance to fungi in vitro. Collectively, these findings provide a basis for further functional studies of *TdPR10* genes, which could be leveraged to enhance stress tolerance in durum wheat.

## 1. Introduction

Due to climatic and environmental variations, plants face challenges such as salinity, cold, drought, heavy metals, and phytopathogen attacks. To overcome these harsh conditions, plants activate complex molecular mechanisms that lead to the production of protective proteins [1]. One of the most important groups of proteins involved in responding to these threats is the pathogenesis-related (PR) proteins [1,2]. PR proteins are sub-divided into 19 groups based on their structural and functional characteristics. PR proteins have crucial roles in regulating plant growth and development under different stressors. Among them, PR10 is one of the most studied groups [3]. PR10 proteins are small, acidic proteins with molecular weights ranging from 16 to 19 kDa. They possess unique three-dimensional β-sheet topologies, enclosed by a compact bipartite framework held together by hydrophobic interactions [4]. PR10 proteins are well known for their role in stress resistance, acting as defense proteins under harmful conditions [5]. These proteins are part of a multigene family, as seen in *Solanum lycopersicon* (45 genes, [6]) and *Hevea brasiliensis* (rubber tree, 132 genes) [7]. Based on phylogenetic studies, PR10 proteins are classified into different subfamilies such as the PR10/Bet v1-like proteins, major latex-like (MLP) proteins, phytohormones binding proteins (PBP), proteins with the enzymatic function that are the (S)-norcoclaurine synthase (NCS) protein, polyketide cyclase-like protein and even monocot PR10 and dicot PR10 [8].

PR10 proteins are related to the major pollen allergen Bet v 1 of white birch (*Betula verrucosa*) [8]. Homologs of Bet v 1 have been identified in many plants, such as cherry (Pru a 1) [9], apple (*Malus domestica*) [10], and carrot (Dau c1) [11]. A highly conserved glycine-rich motif (GXGGXGXXK) is found in all PR10 protein sequences, which enables RNA binding with specific affinity [12]. PR10 proteins are also known for their ribonuclease activity, along with at least eight other distinct enzymatic functions [13]. For instance, PR10 proteins act similarly to neopinone isomerase in opium poppy and to beta-1,3-glucanase in MaPR10 [14,15], as well as other secondary metabolic enzyme functions, such as protein phosphatase inhibitor activity [7]. In *Brassica napus*, the ribonuclease PR10.1 has been shown to enhance germination under salinity stress [9]. Despite the tiny size of PR10 proteins, they can bind small molecules in the hydrophobic cavity formed by the Bet v 1-fold [7]. According to a review by [16], three primary classes of chemicals—cytokinins, flavonoids, and sterols—are associated with potential roles of PR10 proteins in plant metabolite biosynthesis, host defense, and plant growth and development [17,18,19,20]. Moreover, PR10 proteins can interact with hormones and other molecules, such as flavonoids, fatty acids, steroids, gibberellic acid, and cytokinin, due to their hydrophobic cavity [10]. It is thought that the function of PR10 proteins is not always connected to the protein’s contribution to host defense because not all of them have ribonuclease activity [5]. Similarly, the *HcPR10* gene from *Halostachys caspica* was significantly upregulated during development [21]. In addition, *ABR17A* gene exhibits enhancement during germination and in the early seedling growth of *Arabidopsis thaliana* plants [22]. Moreover, PR10 proteins present antimicrobial activities [23]. A *PR10* gene from rice, OsBet v1, exhibits upregulation after infection with the nematode *Meloidogyne graminicola* [24]. In *Escherichia coli*, the production of PmPR10-3.1 from white pine (*Pinus monticola*) inhibited the fungi growth of *Cronartium ribicola* [25]. CaPR-10 isolated from pepper (*Capsicum annuum*) responds against Tobacco Mosaic Virus (TMV) infection with its ribonucleolytic activity [26]. In addition to the biotic stress response, PR10 protein is also involved in abiotic stress tolerance. In rice roots, a *PR10* gene named the *RSOsPR10* gene (root-specific rice PR10) shows both stress resistances, with induction upon blast fungus infection and also after salt and drought stresses [27]. In addition, transgenic *Arabidopsis thaliana* plants overexpress the *PR10* gene from pea (ABR17) and tolerate salinity, heat, and cold stresses [22].

Durum wheat (*Triticum durum* Desf.) is one of the most important cereal crops for human consumption, providing essential bioactive components, proteins, fibers, and carbohydrates [28]. Wheat production and yield are highly dependent on climatic conditions [5]. To date, little is known about the PR10 protein family in durum wheat and its biological function in stress responses.

In this study, we examine the gene and protein features, chromosomal locations, classification, and evolution of PR10 members in durum wheat. We also identify cis-regulatory elements in their promoters and analyze the expression patterns of *TdPR10* genes in response to abiotic stress and hormone treatments. Finally, we explore the role of TdPR10.1 in enhancing LDH protective activity, RNase activity, and antifungal activity in vitro. This study provides a genome-wide analysis of the PR10 family and investigates the expression of *TdPR10* genes under various conditions in durum wheat.

## 2. Results

### 2.1. Genome-Wide Identification and Analysis of T. durum PR10 Family Members

**Figure 1:**
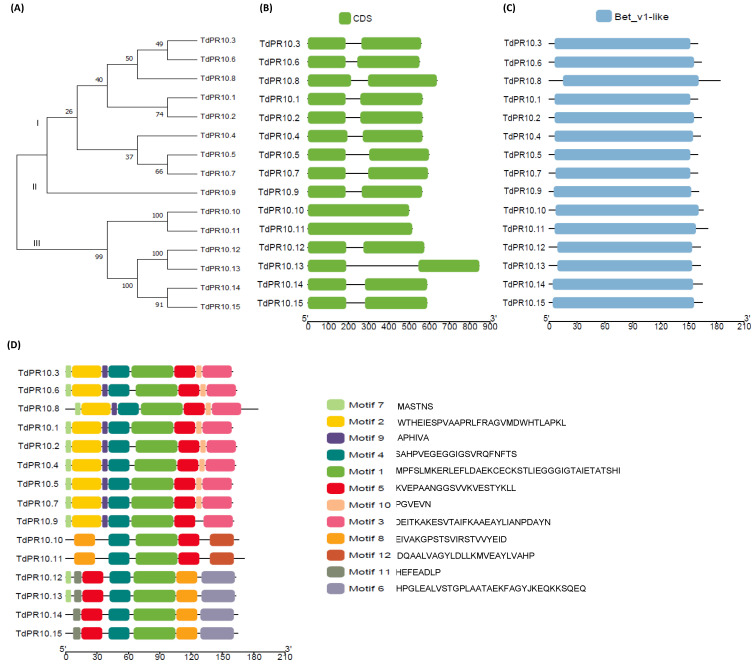
Analysis of *T. durum* PR10 family members. (**A**) Phylogenetic tree of TdPR10 proteins constructed using the maximum likelihood with 1000 bootstraps in MEGA 11 software. (**B**) Structure of *TdPR10* genes. (**C**) Conserved Bet v 1 domains present in PR10 proteins of durum wheat. (**D**) Motifs of TdPR10 proteins visualized using Tbtools II v.070.

Using the isolated PR10.1 protein sequence previously defined by our group as a query, we identified 15 PR10 genes in the durum wheat genome using the EnsemblPlants database. These genes were classified into three major subfamilies (Figure 1A). Two online tools, InterPro and CDD, were employed to confirm the presence of the Bet v 1 conserved domain within the PR10 protein sequences (Figure 1B,C). A high degree of similarity was observed when comparing the Bet v 1 signature logo generated for TdPR10 proteins (Figure 1B) with the Bet v 1 signature motif from Prosite (ID: PS00451) (Figure 1C).

A total of 12 novel motifs were predicted from the amino acid sequences of all 15 TdPR10 proteins from durum wheat using TbTools II v.070 software (Figure 1D). Motif 1 and motif 4 were present in all the putative TdPR10 proteins. The fewest motifs were identified in group II proteins, specifically TdPR10.10 and TdPR10.11 (Figure 1A,D). Additionally, a specific motif arrangement was observed for each protein group. While different types and localizations of motifs were found among the TdPR10 sequences, each phylogenetic tree group exhibited a conserved motif pattern and order. This variability in motif types, locations, and numbers may suggest distinct functions for PR10 proteins in durum wheat. Furthermore, all identified sequences contained the conserved Bet v 1 domain and the glycine-rich P-loop motif (Supplemental Figure S1A). The alignment of amino acid sequences showed high similarity across all PR10 proteins (Supplemental Figure S1A).

Table 1 summarizes the basic properties of TdPR10 proteins. Based on the theoretical isoelectric point (pI) values, which range from 4.59 to 5.94, all deduced PR10 proteins were classified as acidic. The sequence lengths of these proteins vary from 160 to 184 amino acids, while their predicted molecular weights (Mw) range from 16.9 to 19.8 kDa. Among the 15 identified TdPR10 proteins, three exhibited an instability index greater than 40, suggesting that these proteins may be unstable under stress conditions. However, all identified proteins showed a high aliphatic index, indicating that they are likely thermostable. Additionally, the GRAVY (Grand Average of Hydropathicity) index values for the TdPR10 proteins varied, indicating that they could be either hydrophilic or hydrophobic.

### 2.2. Gene Structure and Physical Location of T. durum PR10 Genes

Analyzing the exon and intron distribution patterns provides valuable insights into the structural evolution of the durum wheat *PR10* gene family. The exon–intron organization of the *TdPR10* genes displayed a similar structure, with most genes containing two exons separated by one intron. However, genes from family II (*TdPR10.10* and *TdPR10.11*) were an exception, as they contained only a single exon (Supplemental Figure S1B; Table 1).

**Figure 2:**
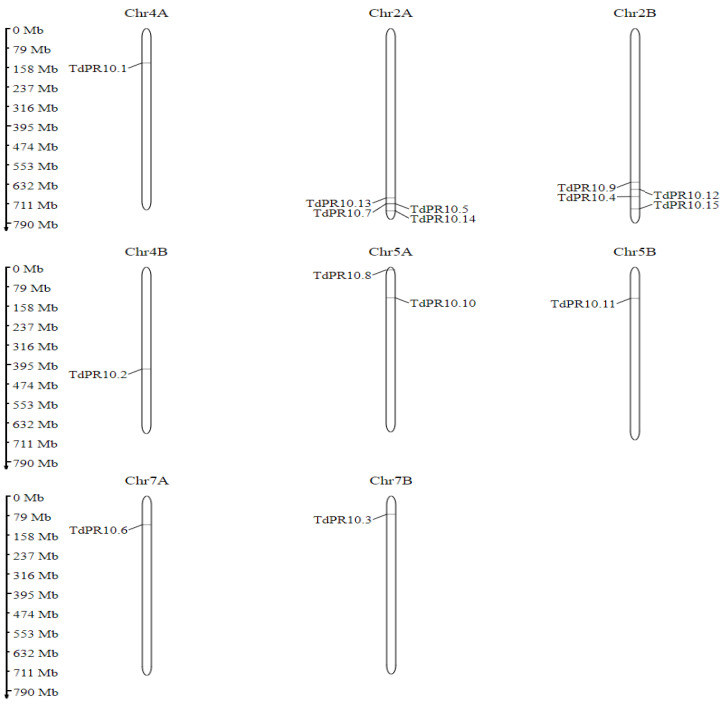
Chromosomal localisation of *TdPR10* genes by using MG2C tool.

To further investigate the distribution of *TdPR10* genes in the durum wheat genome, we found that four genes were localized on chromosomes 2A and 2B, while one gene was located on each of chromosomes 4A, 4B, 5A, 5B, 7A, and 7B (Figure 2). These chromosomes may play a key role in the *TdPR10* gene family and are promising candidates for improving durum wheat traits related to PR10 functional phenotypes.

### 2.3. Phylogenetic Analysis of TdPR10 Proteins

**Figure 3:**
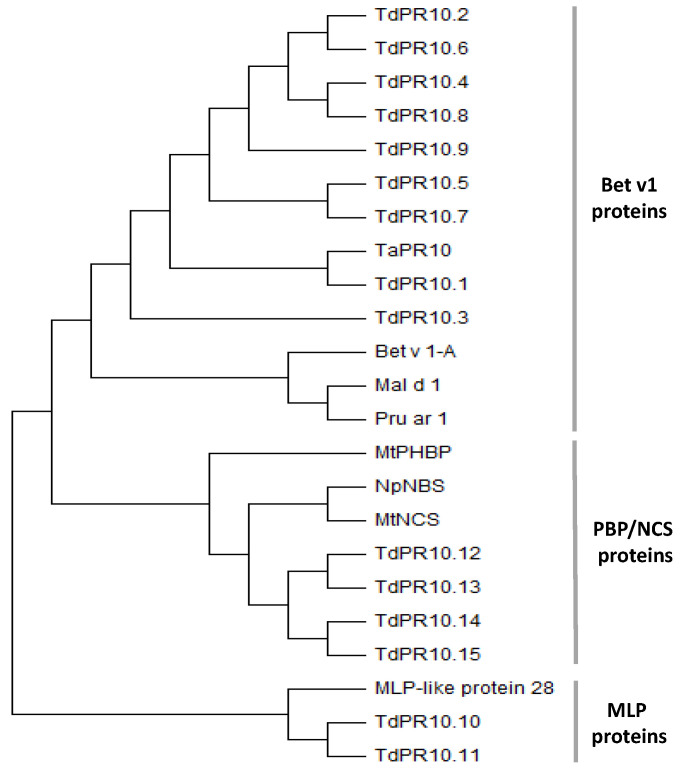
Phylogenetic clustering of TdPR10 proteins alongside well-characterized PR10 proteins: Bet v 1A from birch pollen (*Betula verrucosa*, P15494.2); Mal d 1, the major allergen from *Malus domestica* (NP_001281292.1); NpNBS, norbelladine synthase from *Narcissus pseudonarcissus* (A0A3G5BB24.1); MtNCS, S-norcoclaurine synthase-like protein (KEH30672.1) and MtPHBP, phytohormone-binding protein (G7J032.1) from *Medicago truncatula*; Pru ar 1 from *Prunus armeniaca* (O50001.1); TaPR10, a pathogenesis-related protein 10 from *Triticum aestivum* (ACG68733.1); and MLP-like protein 28 from *Arabidopsis thaliana* (NP_001117579.1). The phylogenetic tree was generated using the maximum likelihood method with 1000 bootstrap replicates in MEGA 11 software.

To classify the identified TdPR10 proteins, a maximum likelihood tree was constructed using a bootstrap of 1000 replicates. The durum wheat PR10 proteins were grouped into three major subfamilies: the Bet v1-like subfamily (TdPR10.1 to TdPR10.9), the PBP/NCS subfamily (TdPR10.12 to TdPR10.15), and the major latex protein (MLP) subfamily, consisting of TdPR10.10 and TdPR10.11 (Figure 3).

In parallel, identity and similarity analyses were performed to predict the molecular functions of TdPR10 proteins (Supplemental Figure S2). Proteins in the Bet v1 group (TdPR10.1 to TdPR10.9) showed a similarity of 42% to 58% to well-known allergens, including Pru av 1 [29], Mal d 1 [10], Bet v 1 [30], Pyr c 1 [31], Api g 1 [32], Dau c 1 [11], Cor a 1 [33], Ara h 8 [34], and Cas s 1 [33]. Additionally, members of this group exhibited 47% to 55% identity with PmPr10-3.1, an antifungal protein resistant to white pine blister rust [35], and PinmIII, which has a role in cold tolerance [36]. Furthermore, comparison of the tomato PR10 protein sequence (Solyc09g090980) [6] with proteins clustered in the Bet v1 and MLP groups revealed 47% to 54% identity, suggesting that these TdPR10 proteins may be involved in virus resistance. Additionally, TdPR10.10 and TdPR10.11 showed close similarity to the rice PBZ1 protein, with 68% and 66% similarity, respectively. Like PBZ1, which functions as an RNase/DNase enzyme [37], TdPR10.10 and TdPR10.11 may play roles in salt tolerance and fungal resistance [38]. TdPR10.12 and TdPR10.13 exhibited 45% similarity to the PR10 protein of soybean, Gly m 4, which is known for its resistance to *Phytophthora sojae* infection [20]. Meanwhile, TdPR10.14 and TdPR10.15 showed approximately 45% similarity to the allergens Bet v 1, Cor a 1, and Cas s 1.

### 2.4. In Silico Subcellular Localisation and Gene Ontology Analysis of the T. durum PR10

**Figure 4:**
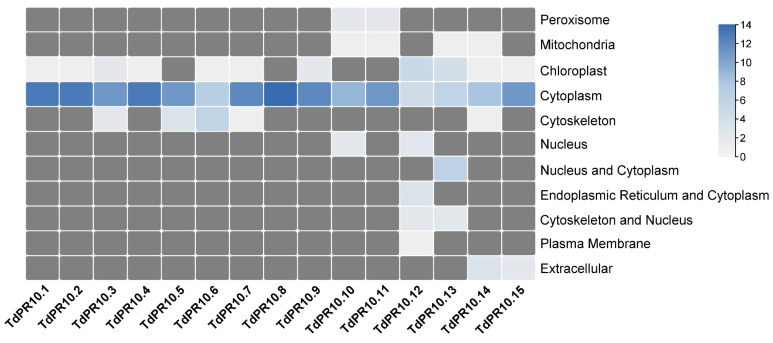
In silico subcellular localisation prediction of TdPR10 proteins using the WoLFPSORT online server.

**Figure 5:**
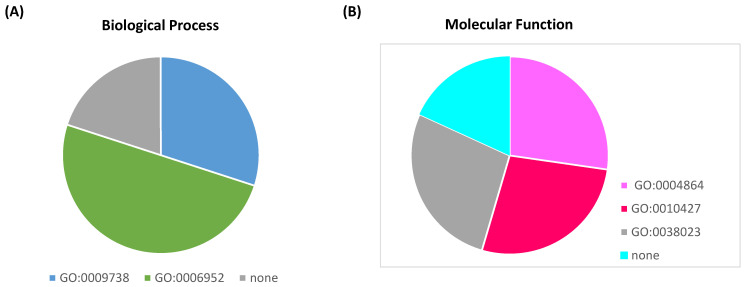
Prediction of biological processes (**A**) and molecular functions (**B**) of PR10 proteins identified in durum wheat based on GO terms in the InterPro database.

The in silico subcellular localization prediction showed that the 15 proteins were predominantly localized in the cytoplasm (Figure 4). In addition, TdPR10.6 was also predicted to be associated with the cytoskeleton, and TdPR10.13 was predicted to be present in the nucleus. The other proteins were localized in the mitochondria, peroxisome, chloroplast, extracellular matrix, and plasma membrane. A total of 15 TdPR10 proteins were analyzed for their reported Gene Ontology (GO) terms in the InterPro database (Figure 5). For all TdPR10 proteins, three main molecular functions were identified: protein phosphatase inhibitor activity (GO:0004864), abscisic acid binding activity (GO:0010427), and signaling receptor activity (GO:0038023). Additionally, two biological processes were associated with these proteins: defense response (GO:0006952) and the abscisic acid-activated signaling pathway (GO:0009738).

### 2.5. Tertiary Structure of TdPR10 Proteins

**Figure 6:**
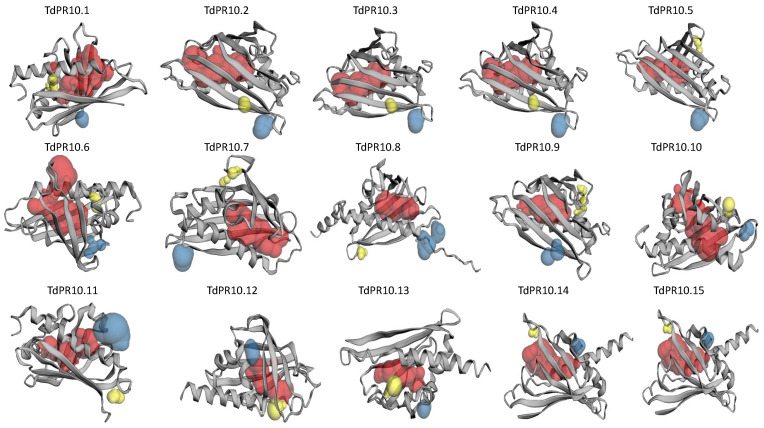
Prediction of TdPR10 3D structure using SWISS-MODEL. Pockets were visualized from largest to smallest using red, blue, and yellow colors, respectively, with the CASTp 3.0 online tool.

The constructed 3D models showed that TdPR10 proteins share a common structure. This structure is organized into a seven-stranded antiparallel β-sheet and three α-helices (Figure 6). Additionally, the models revealed differences in their predicted binding pockets. These findings suggest variability in the substances that may bind to the TdPR10 cavities, indicating different roles for these proteins.

### 2.6. In Silico Analysis of Cis-Elements

**Figure 7:**
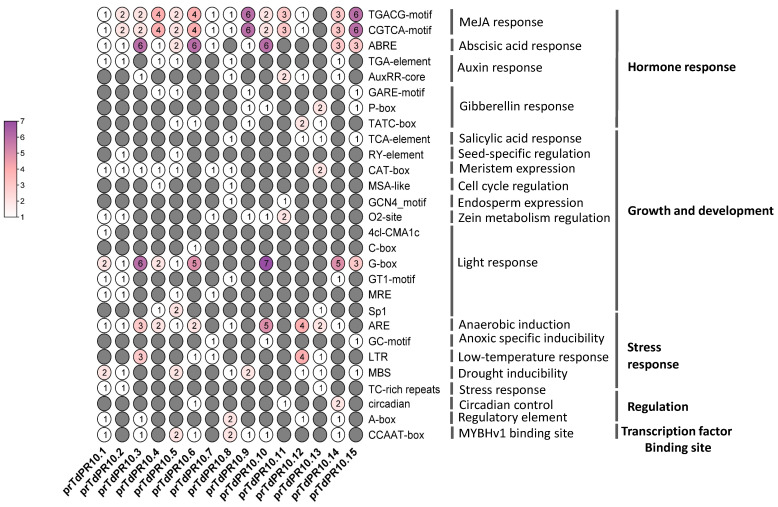
Predicted cis-regulatory elements in the promoter regions (approximately 1500 bp) of *TdPR10* genes were analyzed using the PlantCare and PLACE web tools. The figure was created using TBtools II v.070 software. The putative cis-element numbers are highlighted in different colors, with specific counts marked in each grid.

Cis-elements are involved in gene function and regulation. In the current study, we identified them from the 1.5 kb promoter region of *PR10* genes in durum wheat (Figure 7). Cis-regulatory elements related to hormone responses were found in most of the PR10 promoters. Specifically, MeJA-responsive cis-elements were identified in all prTdPR10 promoters except for prTdPR10.13. Two *PR10* genes, *prTdPR10.14* and *prTdPR10.15*, contained three ABRE elements, while *prTdPR10.3*, *prTdPR10.6*, and *prTdPR10.10* had six ABRE elements. These elements are involved in the abscisic acid response. In contrast, only one or two cis-elements related to auxin and gibberellin responses were present in some of the *PR10* promoter regions. Among the cis-elements implicated in growth and development, G-box elements associated with light responses were detected in most *TdPR10* promoter sequences. Regarding stress responses, ARE elements were present in most *prTdPR10* promoters, with frequencies ranging from 1 to 5 elements. The *TdPR10.10* promoter region possessed the highest number of G-box (6), ABRE (7), and ARE (5) elements. Regulatory cis-elements were found in only a few *PR10* promoters, with a maximum of two elements. Similarly, the MYBHv1 binding site element was present in only 8 out of 15 *TdPR10* promoter regions.

### 2.7. Response of TdPR10 Genes to Several Abiotic Stress and Phytohormones

**Figure 8:**
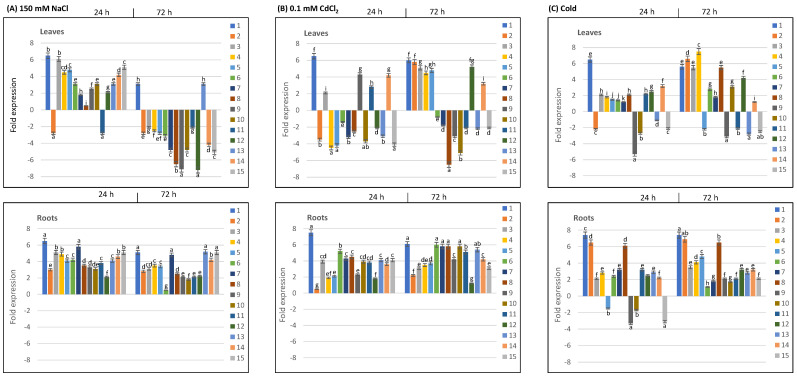
Analysis of the expression profiles of *TdPR10* genes (*TdPR10.1* to *TdPR10.15*) in the leaves and roots of *T. durum* exposed to various abiotic stresses for 24 and 72 h was conducted. Three types of abiotic stress were applied: 150 mM NaCl (**A**), 0.1 mM CdCl_2_ for cadmium toxicity (**B**), and cold at 4 °C (**C**). The expression value of each *TdPR10* gene in the leaves and roots of non-treated plants (control) was set to 1 to calculate the relative expression. Log_2_-transformed values were presented in bar charts. The *TdActin* gene was used as an internal control. Four plants were used per treatment per replicate, and error bars indicate the standard deviation of three biological replicates. In each period of stress application (24 or 72 h), different letters marked on the same bar chart indicate significant differences (*p* < 0.05).

We conducted RT-qPCR studies on the leaves and roots of durum wheat cv. Om Rabiaa, which had been exposed to various abiotic stimuli (salt, cold, and cadmium toxicity) as well as phytohormone treatments (ABA and SA) for 24 and 72 h to investigate the expression profiles of *TdPR10* genes. Our results indicate that the expression of *TdPR10* genes varies depending on the type of stress. Specifically, within 24 h of applying salt stress, the majority of *TdPR10* genes in leaves were upregulated; however, after 72 h, this response diminished or remained unchanged. In contrast, during both salt exposure durations, all *TdPR10* genes were significantly upregulated in roots (Figure 8A). Similarly, under cadmium toxicity, most *TdPR10* genes exhibited positive expression in roots at both time points, although their expression in leaves varied between positive and negative responses (Figure 8B). Notably, during cadmium stress, *TdPR10.1*, *TdPR10.3*, and *TdPR10.14* genes consistently showed upregulation in both leaves and roots. The expression of different *TdPR10* genes varied in response to cold stress (Figure 8C), with some genes consistently upregulated in both leaves and roots during the stress periods. After 24 h of cold stress, *TdPR10.2* exhibited a brief downregulation in leaves, but at 72 h, it increased significantly (by approximately three times). Overall, our results demonstrate that distinct abiotic stresses regulate *TdPR10* genes differently. They also indicate that while responses in leaves varied among *TdPR10* genes, root tissues showed strong induction under all stress conditions.

**Figure 9:**
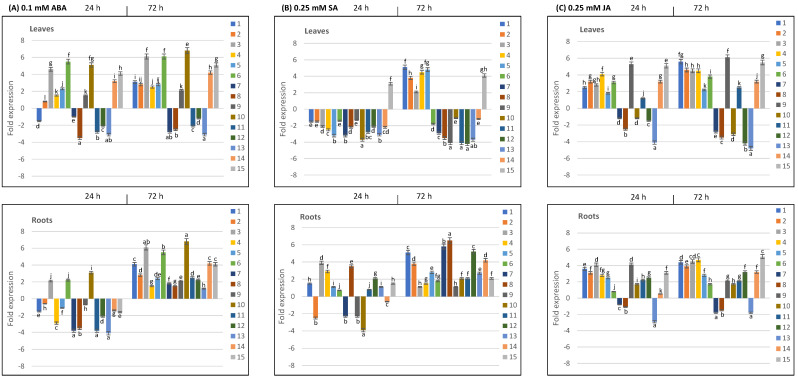
Analysis of the expression profiles of *TdPR10* genes (*TdPR10.1* to *TdPR10.15*) in the leaves and roots of *T. durum* exposed to phytohormones: (**A**) 0.1 mM ABA, (**B**) 0.25 mM SA and (**C**) 0.25 mM JA. The expression value of each *TdPR10* gene in the leaves and roots of non-treated plants (control) was set to 1 to calculate the relative expression. Log_2_-transformed values are presented in bar charts. The *TdActin* gene was used as an internal control. Four plants were used per treatment per replicate, and error bars indicate the standard deviation of three biological replicates. In each period of stress application (24 or 72 h), different letters marked on the same bar chart indicate significant differences (*p* < 0.05).

Likewise, *TdPR10* genes were both up- and downregulated in leaves and roots in response to phytohormone treatments (Figure 9). Notably, during the 24 and 72 h of stress, TdPR10.7, TdPR10.8, TdPR10.12, and TdPR10.13 were consistently downregulated in leaves across all phytohormone treatments. In contrast, leaves treated with ABA exhibited significantly higher expression levels of TdPR10.3, TdPR10.6, and TdPR10.10 (Figure 9A). TdPR10.1 was upregulated in leaves under both ABA and SA treatments, which was particularly evident 72 h after treatment (Figure 9A,B). Furthermore, after receiving hormone treatments for 72 h, the majority of *TdPR10* genes displayed significant expression levels in roots (Figure 9). Among the *TdPR10* genes, *TdPR10.9* and *TdPR10.15* were notably most significantly expressed in leaves treated with JA (Figure 9C). All these results confirm the results of in silico analysis of the cis-elements and highlight the important roles of various *TdPR10* genes in durum wheat’s response to abiotic stress and in regulatory pathways involving plant hormones. Further research will be beneficial to clarify their unique roles in stressful conditions.

### 2.8. Expression of TdPR10.1 Protein in E. coli Strain

The TdPR10.1 cDNA was cloned into the *EcoR*I site of the pGEX4T-1 expression vector. The target protein can be expressed by *E. coli* strain BL21 cells as a fusion protein attached to a GST tag that can be cleaved. When thrombin removes the GST tag, a protein with an N-terminal GSPEF amino acid extension, encoded by the vector, is produced. Size-exclusion chromatography (SEC), thrombin cleavage to eliminate the GST tag, and affinity chromatography were used to purify the tagged proteins. TdPR10 migrates in SDS-PAGE with an apparent molecular mass (MM) of 17 kDa (Supplemental Figure S3).

### 2.9. Functional Characterization of TdPR10.1 In Vitro

#### 2.9.1. TdPR10.1 Protects LDH Activity Under Stress Conditions

**Figure 10:**
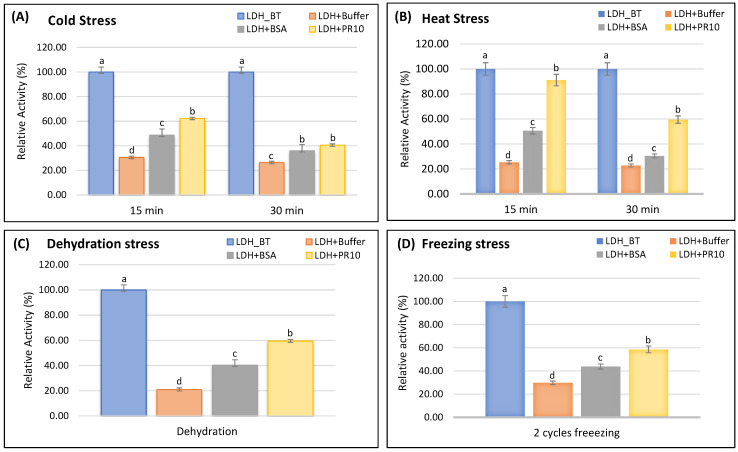
Analysis of the protective role of TdPR10.1 on LDH activity under various stresses in vitro was conducted. LDH activity was measured in solutions mixed with sucrose (buffer), BSA (buffer + BSA), or the purified His-tagged TdPR10.1 protein (buffer + TdPR10.1) under cold (**A**), heat (**B**), dehydration (**C**), and freezing (**D**) conditions. LDH activity before treatment (BT) was taken as 100%. Error bars indicate the standard deviation of four biological replicates. In each period of stress application, different letters marked on the same bar chart indicate significant differences (*p* < 0.05).

We tested the ability of TdPR10.1 to prevent the loss of LDH activity after heating, dehydration, cold, and freezing. We compared the effects of TdPR10.1 with those of BSA as a non-specific protective agent and with LDH treated in buffer without additional protein. We investigated TdPR10.1’s impact on LDH enzyme activity under cold and heat stresses. The LDH enzyme was combined with either BSA or a TdPR10.1-free phosphate buffer. Activity was assessed immediately after aliquots were incubated at 0 °C and 43 °C for various exposure durations, ranging from 0 to 30 min (Figure 10A,B). The LDH activity rapidly drops at 0 °C, reaching just 26.7% of its relative activity after 30 min of incubation. However, LDH inactivation is significantly reduced in the presence of BSA or TdPR10.1. After 30 min of incubation at 0 °C with BSA, its relative activity was approximately 37.38%, while for TdPR10.1, it was around 40.75% (Figure 10A). The same results were obtained under heat stress. In fact, the enzyme’s activity rapidly declined at 43 °C, reaching just 22.1% of its relative activity after 30 min of incubation. However, LDH inactivation is greatly reduced in the presence of BSA or TdPR10.1. After 30 min of incubation at 43 °C, the profile of LDH activity in the presence of TdPR10.1 was significantly higher than that of BSA, according to our data. In contrast to the activity in the presence of BSA, which was approximately 30.38%, TdPR10.1 sustained an LDH activity of 60.14% for up to 30 min (Figure 10B). These experimental results demonstrate that TdPR10.1 can shield LDH from heat effects in vitro, indicating a potential protective function during heat stress. Following hydration and dehydration, LDH activity was around 20.8% of the starting value. At all mass ratios, TdPR10.1 provided greater stability to the enzyme, reaching 60%, compared to BSA, which reached 40% (Figure 10C). After two cycles of freezing and thawing, LDH activity decreased to 29.82%, while BSA activity increased to 43.86%. In contrast, TdPR10.1 increased LDH activity to 58.60% (Figure 10D). These results confirm that TdPR10.1 offered a greater degree of protection to LDH than BSA after various stress treatments, indicating that the TdPR10.1 protein has protective activity beyond the non-specific effects of BSA (Figure 10).

#### 2.9.2. TdPR10.1 Improves RNase Activity Under Stress Conditions

**Figure 11:**
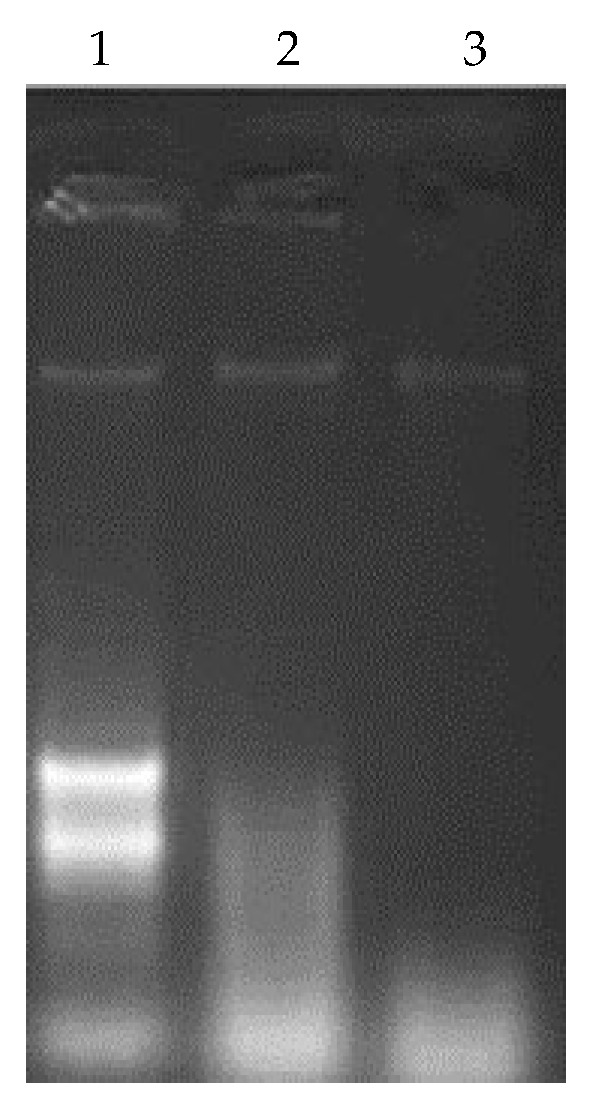
Ribonuclease activity of recombinant TdPR10.1 purified protein was assessed using 30 μg of total RNA extracted from ‘Om Rabiaa’ durum wheat leaves. Gel electrophoresis was performed to separate the RNAs on a 1.2% agarose gel. (1) RNA from ‘Om Rabiaa’; (2) RNA after 1 h of incubation with TdPR10.1; (3) RNA after 4 h of incubation with TdPR10.1.

We tested the potential role of the TdPR10.1 protein in degrading RNA. As shown in Figure 11, RNA alone in nuclease-free water remained intact, whereas RNA with 10 µg of TdPR10.1 was partially degraded after 1 h and completely degraded after 4 h. The results from the RNase activity assays confirm that the TdPR10.1 protein exhibits ribonuclease activity.

#### 2.9.3. Antifungal Activity of TdPR10.1 Protein

To evaluate the inhibitory effect of the TdPR10.1 protein against the tested fungi, the minimum inhibitory concentrations (MICs) and minimum fungicidal concentrations (MFCs) were determined and are presented in Table 2. Interestingly, the MIC and MFC values indicate that the protein has a fungicidal effect against *F. graminearum* and *A. niger*, as the MFC/MIC ratio (500/500 = 1) was less than 4. However, for *F. oxysporum*, *F. culmorum*, and *B. cinerea*, the MFC/MIC ratio was greater than 1 (Table 2).

## 3. Discussion

Tetraploid wheat (*Triticum turgidum* subsp. *durum* (Desf.)) is one of the most economically important cereal crops worldwide. During its lifecycle, various atmospheric and pathogenic stresses negatively affect its growth. In response, plants employ molecular mechanisms to defend against these circumstances. Pathogenesis-related protein 10 (PR10) is a well-recognized protein known for its contribution to plant defense across several species. In the current study, we performed a genome-wide analysis of PR10 in durum wheat. We identified 15 *TdPR10* genes clustered into three major subfamilies (Figure 3). The Bet v 1 group possesses the highest number of TdPR10 members, while the MLP group contains only two proteins (TdPR10.10 and TdPR10.11). Zhang et al. [39] confirm that MLP proteins have a smaller gene number in monocots compared to dicot plants.

Recently, it has been shown that the genome of *H. brasiliensis* (rubber tree) contains 132 *PR10*-encoding genes clustered into two major groups: the major allergen Pru ar 1-like proteins and the major latex protein (MLP)-like proteins. Additionally, three minor groups (three phytohormone-binding proteins (PhBPs), two norbelladine synthase proteins, and LP423/uncharacterized proteins) were identified within the two major groups. Most of these genes are located on chromosome 15. The corresponding PR10 proteins are small, acidic proteins with molecular weights ranging from 11 to 41.52 kDa that lack any signal peptide, in contrast to other PR proteins such as PR-1 [40]. Predicted protein functions indicate that HbPR10 may be involved in protein phosphatase inhibitor activity, abscisic acid binding activity, and signaling receptor activity. These proteins are implicated in plant defense and responses to ABA-activated signaling pathways [7]. In grape, identified PR10 proteins were classified into five groups: ABA receptors, MLP-like proteins, major allergen Pru av 1 protein/STH-2 proteins, S-norcoclaurine synthase (NCS)-like proteins, and uncharacterized proteins [39]. Moreover, the putative PR10 proteins identified in durum wheat were closely related to allergens such as Bet v 1 [41], Pru av 1 [29], Mal d 1 [10], and Cas s 1 [33], suggesting their potential functions as allergens. However, a myriad of allergens play crucial roles in plant defense against abiotic and biotic stresses [42]. For instance, the allergen Gly m 4 from *Glycine max* enhances resistance to *Phytophthora sojae* infection [20]. The major allergen from apple (*Malus domestica*), Mal d 1, exhibits tolerance against *Alternaria alternata* infection [43] and also against stressful environmental challenges (chemical exposure, wounding) [44]. These data allow us to formulate further hypotheses regarding the possible roles of TdPR10 proteins. Furthermore, the P-loop motif of the nucleotide binding proteins, which is involved in binding, catalysis, recognition and regulation of activity of the substrate [45], was harbored by all the putative TdPR10 proteins. Thus, motif 1 present in all the TdPR10 sequences (Figure 1D) is associated with the glycine rich P-loop motif (Supplemental Figure S1). This affirmation led us to suggest the role of TdPR10 wheat plants. Bet V 1 signatures were presented by motif 1 (which was harbored in all PR 10 sequences) and motif 5 (group I and II) or motif 8 (group III). These results are explained by the different classifications of TdPR10 proteins (Figure 3). On the other side, a unique motif arrangement was strongly associated with one protein subgroup, based the classification of TdPR10 proteins in the phylogenetic tree (Figure 1A,D).

Based on the predicted coding sequences and their corresponding genomic sequence results, each TdPR10 subfamily shares a common exon-intron distribution, as well as a similar arrangement and localization of conserved motifs. Thus, during their evolution, variations in gene structures may have occurred. In durum wheat, most PR10 genes from different plant species contain two exons and one intron, while a few possess a unique exon [6,46]. In contrast, in *Vitis vinifera*, two *VvMLP* genes have three exons [39].

Analysis of the physicochemical characteristics of PR10 proteins from various species yields similar results. Almost all PR10 proteins have a pI of less than 7. For instance, as observed in durum wheat, all PR10 proteins in cashew nuts are acidic [47], while only 7 out of 45 in tomato [6] and 3 out of 34 in common bean [46] are basic. In this study, in silico analysis shows that the deduced PR10 proteins of wheat are located in the cytoplasm, similar to their orthologues in tomato plants [6]. Moreover, TdPR10.13 is potentially localized in both the cytoplasm and nucleus, akin to PvMLP2 [46] and HcPR10 [48].

Different localizations of PR10 proteins have been reported in previous studies. For instance, VpPR10.2 proteins were dynamically distributed inside or outside of host cells upon the invasion of the oomycete *Plasmopara viticola* [19]. Gly m 4l proteins were found at the cell membrane in *Arabidopsis* protoplast cells [20]. The HcPR10 protein was present in both the nucleus and cytoplasm during plant development [48]. These findings provide insights into the roles of PR10 proteins in plant defense and stress tolerance and will aid our future analyses to advance our understanding of the roles of PR10 proteins in durum wheat plants.

To gain insights into the role of *TdPR10* genes, we analyzed the predicted cis-elements in their promoter regions (Figure 7). The findings indicated that cis-regulatory elements related to hormonal signaling and stress responses are prevalent in most TdPR10 promoters. Among these, the prTdPR10.1 promoter contains the highest number of cis-elements associated with hormone signaling, growth and development, and stress responses. Previous studies have similarly highlighted the involvement of *PR10* genes in both biotic and abiotic stress responses, as well as in phytohormone signaling [39,46]. Therefore, we suggest that the expression of *PR10* genes is induced in response to various environmental conditions. Furthermore, the diversity of these cis-regulatory mechanisms may contribute to the evolution of gene expression [49].

Stressors such as salt, cold, and metal toxicity induce complex physiological, biochemical, and molecular responses in plants [7,44]. Our RT-qPCR data (Figure 8 and Figure 9) demonstrate that *TdPR10* genes exhibit distinct expression patterns in response to abiotic stress and phytohormone treatments. Notably, after 24 h of exposure to these abiotic stresses, the *TdPR10.1*, *TdPR10.3*, and *TdPR10.14* genes were significantly activated in leaves (Figure 8), indicating their critical role in durum wheat’s adaptation to various environmental conditions. Similar findings regarding the induction of PR10 expression by hormones and abiotic stressors have been reported in different plant species. For instance, roots being subjected to salt, dehydration, and fungal infections induces RsOsPR10 [30]. Additionally, salicylic acid (SA) and copper stress activate SsPR10 in *Solanum surattense* and ZmPR10 in maize [46,47]. In common bean roots, PvPR1 and PvPR2 proteins are induced by copper stress [48], while jasmonate (JA) signaling activates many *PR10* genes [19,30].

Quantitative real-time PCR (RT-qPCR) analyses showed that 10 *PR10* genes are involved in roots under cold and NaCl stresses during 72 h of stress application, with the exception of PR10.2, PR10.9, and PR10.10. In leaves, PR10.1 was downregulated during the 72 h following NaCl stress application, whereas other genes were upregulated after 24 h of stress and subsequently downregulated at 72 h. Interestingly, the majority of PR10 genes were downregulated in leaves after hormonal application (SA, ABA) [46]. Moreover, *PvPR10* genes in leaves are upregulated by exogenous JA and ethylene precursor (ETP) application, suggesting their involvement in JA/ET signaling pathways [37]. Recent investigations have demonstrated that PR10/Bet v1 proteins play critical roles during the biotrophic phase of common bean infection cycles through their interactions with the ethylene (ET), SA, and JA pathways [49].

In our study, we observed that exogenous treatments with ABA, SA, or JA significantly increased TdPR10.15 expression in leaves, while many *TdPR10* genes exhibited strong expression in roots (Figure 9). Recently, 24 *SsPR10* family genes were identified in the *Saccharum spontaneum* genome, subdivided into two subfamilies: IPR10 (SsIPR10-1–14) and NCS (SsNCS-1–10). Bioinformatics analyses indicated that six and two sets of SsPR10 underwent tandem and fragmental duplication events, respectively. Foliar application of exogenous SA in two sugarcane cultivars (LCP85-384 and ROC20) resulted in significant differences in the expression profiles of several *PR10* genes. For instance, SsPR10-1, -2, -4, -7, -11, and -12 were upregulated in LCP85-384 at 12 h post-treatment (hpt) but downregulated by 81.3% in ROC20 at 24 hpt compared to the control [50].

To date, cryoprotective activity has been tested in various cold-induced proteins, such as COR15 in *Arabidopsis* [51] and CAP85 in spinach [52], using lactate dehydrogenase (LDH) as a freeze-labile model enzyme. However, these proteins belong to the late embryogenesis abundant (LEA) protein family [53]. To our knowledge, AHCSP33, a PR5 protein from groundnut, is the only example within the PR protein family that has demonstrated in vitro cryoprotective activity against LDH [54]. Additionally, WAP18, a member of the PR-10/Bet v 1 protein family in mulberry, has also exhibited cryoprotective activity against the freeze-labile enzyme LDH [55]. These findings suggest that WAP18 may play a crucial role in enhancing freezing tolerance in mulberry trees during winter [55]. In our study, TdPR10.1 demonstrated protective effects against heat, cold, freezing, and dehydration, showing more significant activity than BSA, a well-known protective protein [56]. Our data confirm that TdPR10.1 effectively preserves LDH activity against enzymatic inactivation caused by these stress treatments.

In addition to their involvement in the signaling pathways of defense genes, PR10 proteins are known for their ribonucleolytic activity, which allows them to cleave the RNA of invading pathogens [4]. During pathogen infection, the RNase activity of PR10 proteins can exert a cytotoxic effect on cells and inhibit pathogen growth by degrading pathogen RNA [6,57,58,59]. This inhibition primarily occurs through the penetration of ribonucleases into the pathogen, followed by the phosphorylation of PR10 proteins, leading to the degradation of pathogenic cell RNAs [60]. A novel PR10 protein, SaPR10.1, was isolated from *Saccharum arundinaceum*. SaPR10.1 is a small, hydrophilic, acidic protein that lacks the P-loop motif typically associated with RNase activity in other PR10 proteins. Its complex 2D structure suggests that SaPR10.1 may bind to multiple molecules of trans-zeatin, which differs from other orthologs. The authors propose that SaPR10.1 could play a role in the more efficient depletion of free trans-zeatin [61]. In rice, the RNase activity of JIOsPR10 was found to be abolished following treatment with DTT in a native in-gel assay. Additionally, the substitution of specific serine residues (C81S, C83S, C81/83S) significantly decreased the RNase activity of the C83S mutant, highlighting the critical role of disulfide bonds between cysteine residues in PR-10 proteins. These bonds may be important for the constitutive self-defense mechanisms in plants against both biotic and abiotic stresses [37]. While several PR10 proteins exhibit RNase activity, it is not considered a universal characteristic [62]. RNase activity is particularly relevant under biotic and abiotic stress conditions, as these proteins participate in plant hypersensitive response (HR) signaling, programmed cell death, and apoptosis processes [18,63].

Additionally, PR10 proteins can interact with plant hormones such as ABA, JA, IAA, ET, and SA, which are involved in hormone-mediated signaling pathways that mitigate damage caused by biotic and abiotic stress [4,64]. For example, in plants infected with *Verticillium dahliae*, *PR10* genes were found to be upregulated following an expression profile investigation in the leaves, roots, and stems of strawberry plants [65]. The induction of several phytohormones, including ABA, SA, JA, and GA, was observed during the early stages of plant–pathogen interactions. In contrast, only two hormones, IAA and JA, were induced in the roots, and this occurred during the later stages of infection [65].

There is substantial evidence supporting the general efficacy of PR10 proteins against a range of phytopathogens, including fungi, bacteria, and viruses [4,16]. Furthermore, one study highlighted the protease inhibitory activity of PR10 proteins against the root-knot nematode *Meloidogyne incognita* [66]. Regarding the activity of PR10 proteins against pathogens, although the mechanisms are not fully elucidated, these enzymes are believed to be associated with endogenous cytokinin (CK) concentrations and may participate in negative feedback regulation. CK plays a crucial role in modulating plant immunity, directly influencing the plant’s defense response to various pathogens [4,67,68].

In our study, we found that the TdPR10.1 protein exhibits antifungal activity against *Fusarium graminearum* and *Botrytis cinerea*, with both minimum inhibitory concentration (MIC) and minimum fungicidal concentration (MFC) values around 500 µg/mL. Since the MFC/MIC ratio equals 1 for these fungi, TdPR10.1 is classified as a fungicidal agent. Furthermore, the same ratio of 1 was observed for *Aspergillus niger* and *Fusarium graminearum*, indicating that the minimum concentration required to inhibit fungal growth is the same as that needed to kill the fungi completely. This suggests that TdPR10.1 is an effective fungicidal agent.

## 4. Materials and Methods

### 4.1. Identification of PR10 Family Members of T. durum

Members of the PR10 family in the *Triticum durum* (Svevo.v1) genome were identified using a BLAST search, with our previously identified TdPR10.1 serving as the query. This search utilized the Ensembl Plants database (https://plants.ensembl.org/index.html (accessed on 20 February 2024)). The presence of the conserved domain characteristic of PR proteins, specifically the Bet v 1 domain (PF00407), was verified in the retrieved sequences through InterPro (https://www.ebi.ac.uk/interpro/ (accessed on 20 February 2024)) and CD-search (https://www.ncbi.nlm.nih.gov/Structure/cdd/wrpsb.cgi (accessed on 22 February 2024)) [69]. Any redundant sequences were manually removed from the dataset.

### 4.2. Characterization of T. durum PR10 Family Members

The conserved motifs and the Bet v 1 domain (PF00407) of the *Triticum durum* PR10 family members were identified using TBtools-II v 2.070 software [70]. Gene structures were visualized with the same program after obtaining GFF3 files for the *TdPR10* genes from the Ensembl Plants website (https://plants.ensembl.org/index.html (accessed on 22 February 2024)). Chromosomal gene mapping of the *T. durum* PR10 family was conducted using the MG2C v2.1 online tool (http://mg2c.iask.in/mg2c_v2.1/ (accessed on 24 February 2024)). Physicochemical parameters, including molecular weight, theoretical pI, and instability index for the members of the *T. durum* PR10 family, were determined using the ProtParam tool (https://web.expasy.org/protparam/ (accessed on 25 February 2024)). Additionally, the in silico subcellular localization of these proteins was predicted using the WolfPsort subcellular localization predictor (https://wolfpsort.hgc.jp/ (accessed on 25 February 2024)) [71]. Biological processes and molecular functions of the TdPR10 proteins were retrieved from the InterPro database (https://www.ebi.ac.uk/interpro/ (accessed on 26 February 2024)).

### 4.3. Phylogenetic Relationships Analysis of TdPR10

A multiple sequence alignment of TdPR10 proteins was performed using the MUSCLE algorithm in MEGA 11 software [72] and visualized with GeneDoc v2.7 [73]. The PR10 protein family in *Triticum durum* was clustered into a phylogenetic tree alongside known PR10 proteins from various plant species using the maximum likelihood method with 1000 replicates in MEGA 11 [72]. Furthermore, to determine the percentage of identity between proteins, we utilized TBtools-II v 2.070 software [70].

### 4.4. Prediction of Tridimensional Structure of TdPR10 Proteins

The 3D structures of TdPR10 proteins were predicted using the SWISS-MODEL server [74]. To visualize the molecular pockets of these proteins, we employed the CASTp 3.0 online server (http://sts.bioe.uic.edu/castp/calculation.html (accessed on 27 February 2024)).

### 4.5. Analysis of Cis-Acting Regulatory Elements in the TdPR10 Promoter Regions

TdPR10 promoter sequences extending 1.5 Kb upstream of the start codons were retrieved from the EnsemblPlants database (https://plants.ensembl.org/index.html (accessed on 2 March 2024)). Each sequence was then analyzed using the PlantCare web tool (https://bioinformatics.psb.ugent.be/webtools/plantcare/html/ (accessed on 2 March 2024)) [75].

### 4.6. Plant Material and Stress Treatments

Seeds of durum wheat, cv. Om Rabiaa, were used as plant material and surface-sterilized with 95% alcohol followed by treatment with 0.2% mercuric chloride [76]. The seeds were then germinated in a mixture of peat and perlite (2:1 ratio) in a glasshouse maintained at day and night temperatures of 24 °C and 18 °C, respectively, with relative humidity ranging from 60% to 70%. Two-week-old seedlings were subjected to salt stress by irrigation with a 150 mM NaCl solution [77] or cadmium chloride (CdCl_2_) at a concentration of 0.1 mM [78]. Cold stress was induced by transferring the seedlings to 4 °C. Phytohormones, including jasmonate (JA; 0.25 mM) and salicylic acid (SA; 0.25 mM), were applied via foliar spraying as described by [79]. Additionally, the seedlings were treated with abscisic acid (ABA; 0.1 mM) [80]. Control seedlings were maintained under normal conditions without stress. After 24 and 72 h of stress application, leaf and root tissues were collected from both treated and control plants, immediately frozen in liquid nitrogen, and stored at −80 °C for total RNA extraction. Three separate biological replicates were performed, with four plants used per replicate.

### 4.7. RNA Isolation and Real-Time Quantitative PCR

Total RNA was isolated from plant tissues using TRIzol reagent (Thermo Fisher Scientific, Waltham, MA, USA) with the modifications to the method described by [62]. The extracted RNA was treated with 1 U of RNase-free DNase (Thermo Fisher Scientific, Waltham, MA, USA) for 10 min at 37 °C to degrade any residual genomic DNA. Subsequently, the RNA was used for the synthesis of first strand cDNA using M-MLV reverse transcriptase (Invitrogen) and Oligo-dT(18 mer). For RT-qPCR experiments, 3 μL of synthetic cDNA (equivalent to 40 ng) was mixed with 5 μL of Maxima SYBR Green qPCR Master Mix (2×) (Thermo Fisher Scientific, Waltham, MA, USA), 0.5 μL of each primer (10 μM), resulting in a total mixture volume of 10 μL. PCR reactions were performed using the CFX96 real-time PCR detection system (Bio-Rad, Hercules, CA, USA) as described by [81]. The quantitative RT-qPCR reactions began with initial denaturation at 94 °C for 10 min, followed by 45 cycles at 94 °C for 10 s, 60 °C for 10 s, and 72 °C for 15 s. The reference gene TdActin was used to normalize gene expression levels. Primer sequences for RT-qPCR were designed for gene specificity using Primer3 web v4.1.0 (https://primer3.ut.ee/ (accessed on 15 July 2024)) (see Table S1). Relative expression levels were calculated using the 2−ΔΔCT method [82]. Each sample was analyzed in triplicate (technical replication), and each experimental condition was performed in triplicate (biological replication).

### 4.8. Production and Purification of TdPR10.1 Protein

To construct the expression vector pGEX-TdPR10.1, specific primers with restriction enzyme sites (*EcoR*I) were designed to ensure in-frame cloning: PR10_EcoI_Fw (5′-GAATTCATGGCATCTTCCAAGAGT-3′) and PR10_NotI_Rev (5′-GAATTCGGCTTCGGCGTCAAG-3′). The amplified products were cloned into the pGEX vector at the *EcoR*I site to express the pGEX-TdPR10.1 fusion protein. The E. coli strain BL21 was used for recombinant protein expression. Cultures were grown overnight in LB medium supplemented with 100 µg/mL ampicillin. An aliquot of the overnight culture was diluted 1:50 with 1 L of LB medium and grown at 28 °C. When the optical density at 600 nm (OD) reached 1, isopropyl β-D-thiogalactopyranoside (IPTG) was added to a final concentration of 1 mM. The induced cells were harvested, washed, and collected by centrifugation at 6000 rpm for 20 min at 4 °C. The resulting pellets were frozen at −20 °C. Each bacterial pellet was resuspended in an equal volume of active aluminum oxide and ground on ice. Subsequently, 35 mL of lysis buffer (20 mM Tris-HCl, pH 8; 100 mM NaCl; 1 mM PMSF; 0.5% NP-40) was added and mixed well. The lysates were clarified by centrifugation at 6000 rpm for 20 min at 4 °C. The clarified supernatant was then incubated overnight with 1 mL of glutathione Sepharose 4B resin (GE Healthcare, Chicago, IL, USA), which had been previously equilibrated with 1X PBS, for 1 h with gentle shaking at 4 °C. The resin was washed three times with 1X PBS. To remove the GST-tag, 1 mL of Phosphate Buffered Saline (PBS, pH 7.3) containing 5 U of bovine thrombin (Calbiochem, Darmstadt, Germany) was added to the resin. After a 16-h incubation at 22 °C, the non-retained fraction was recovered by pelleting the resin. To ensure optimal recovery of the protein of interest, the resin was washed with 1 mL of PBS, yielding a final volume of 2 mL. The presence of the protein was confirmed by SDS-PAGE, and protein concentrations were determined using a spectrophotometer at 595 nm with the Bradford protein assay.

### 4.9. In Vitro Analysis of TdPR10 Protein

#### 4.9.1. LDH Protective Assay

A solution of freeze-labile lactate dehydrogenase (LDH) enzyme (EC 1.1.1.27, rabbit muscle LDH) from Sigma (Tokyo, Japan) was prepared at a concentration of 20 μg/mL in 10 mM sodium phosphate, pH 7.4. This LDH solution was mixed with an equal volume of buffer containing either 20 μg/mL of bovine serum albumin (BSA) or TdPR10-1. The samples were then subjected to heat stress, cold stress, freezing stress, and dehydration stress treatments. To determine LDH activity, 20 μL of the LDH mixture was diluted to 1 mL with assay buffer (10 mM sodium phosphate, pH 7.4, 2 mM NADH, and 10 mM pyruvic acid). The oxidation of NADH was monitored by measuring the absorbance at 340 nm (A340) over 3 min, during which the reaction rate remained linear. The rate of absorbance change was used to calculate the activity using the formula ΔDO/min × 8095 = U/L\Delta DO/min\times 8095 = U/LΔDO/min × 8095 = U/L (Biomaghreb kit). All samples were assayed in triplicate.

#### 4.9.2. Ribonuclease Activity

The RNase activity of the purified recombinant TdPR10.1 protein was assessed by incubating the reaction mixtures at 37 °C for 1 to 4 h, following the method described in [83]. To evaluate the RNA degradation activity, 20 µL of reaction mixture was prepared, containing 10 µg of total RNA extracted from durum wheat ‘cv. Om Rabiaa’ and 30 µg of TdPR10.1 protein. The reaction mixture was then extracted with phenol-chloroform (1:1), and the aqueous layer was analyzed on a 1.2% agarose gel. RNA was visualized under UV light.

#### 4.9.3. Antifungal Activity Tests/Evaluation of Minimal Inhibitory Concentration (MIC) and Minimal Fungicidal Concentration (MFC)

The minimum inhibitory concentration (MIC) of the TdPR10.1 protein was determined against various phytopathogenic fungi, namely *Fusarium oxysporum*, *Fusarium culmorum*, *Fusarium graminearum*, *Aspergillus niger*, and *Botrytis cinerea*, using the broth microdilution method in 96-well microplates, as described in [84]. A twofold serial dilution of the protein was prepared in the microplate wells, ranging from 1.95 to 500 µg/mL. Subsequently, 10 μL of a fungal spore solution (10^5^ spores/mL) was added to each well, and the plates were incubated at 25 °C for 72 h. Wells containing fungal spores without TdPR10.1 served as positive controls. The MIC was defined as the lowest concentration of the protein that exhibited no visible growth of the tested microorganisms after incubation.

For the minimum fungicidal concentration (MFC) evaluation, 10 μL was taken from each well showing fungal proliferation, plated on PDA agar, and incubated at 25 °C for 72 h. The lowest concentration that yielded no growth on the agar was considered the MFC, indicating that >99.9% of the original inoculum was killed. The experiments were repeated three times to ensure reliability.

### 4.10. Statistical Analysis

Statistical analysis of the results was conducted using SPSS version 20, employing the ANOVA method. Means were compared using Tukey’s HSD test, with different letters indicating significant differences (*p* < 0.05).

## 5. Conclusions

In the current study, we identified 15 genes belonging to the PR10 family in the durum wheat genome. Using phylogenetic analysis, we grouped these genes into three major subfamilies. We further investigated their evolution by examining their structure, relationships, chromosomal arrangement, synteny, and repeat patterns. Our results revealed a significant increase in the activity of several *TdPR10* genes in the leaves and roots under stress, indicating their involvement in various metabolic pathways in durum wheat. Moreover, TdPR10.1 demonstrated protective effects on lactate dehydrogenase (LDH) and exhibited RNase activity in vitro, confirming its role as a pathogenesis-related enzyme. Finally, the TdPR10.1 protein displayed fungicidal effects against *A. niger* and *F. graminearum*, while serving as a growth inhibitor for *F. oxysporum* and *B. cinerea*. However, further research is needed to elucidate the functions of these *TdPR10* genes and their potential contributions to enhancing crop stress tolerance.

## Figures and Tables

**Table 1:** Physico-chemical properties of TdPR10 proteins using the Protparam online tool.

Gene Name	Transcript ID	Protein Length (aa)	Molecular Weight (Da)	Theoretical pI:	The Instability Index (II)	Aliphatic Index	GRAVY
*TdPR10.1*	MK570865.1	160	17,113.47	5.19	41.79 unstable	83.62	0.011
*TdPR10.2*	TRITD4Bv1G117510.2	164	17,469.88	5.07	41.23 unstable	87.50	0.016
*TdPR10.3*	TRITD7Bv1G026790.1	160	17,078.41	5.19	38.27 stable	86.00	−0.034
*TdPR10.4*	TRITD2Bv1G226310.3	163	17,372.76	5.94	31.33 stable	81.47	−0.067
*TdPR10.5*	TRITD2Av1G263640.1	160	16,990.30	5.51	37.65 stable	84.25	−0.027
*TdPR10.6*	TRITD7Av1G052120.1	164	17,538.95	5.06	37.12 stable	86.34	−0.024
*TdPR10.7*	TRITD2Av1G263650.1	160	17,080.38	5.57	39.16 stable	83.00	−0.091
*TdPR10.8*	TRITD5Av1G005340.2	184	19,821.80	5.22	41.08 unstable	91.85	0.081
*TdPR10.9*	TRITD2Bv1G209060.1	161	17,015.27	5.08	34.43 stable	82.42	0.014
*TdPR10.10*	TRITD5Av1G049040.1	166	17,127.53	4.62	30.23 stable	94.16	0.213
*TdPR10.11*	TRITD5Bv1G045600.1	171	17,701.17	4.70	23.12 stable	97.08	0.184
*TdPR10.12*	TRITD2Bv1G217530.1	163	17,983.73	4.98	27.50 stable	101.78	−0.001
*TdPR10.13*	TRITD2Av1G253700.1	163	18,047.77	4.91	35.75 stable	100.55	−0.055
*TdPR10.14*	TRITD2Av1G278210.1	165	17,996.20	4.59	35.79 stable	88.55	−0.255
*TdPR10.15*	TRITD2Bv1G242310.1	165	18,180.48	4.84	25.76 stable	92.06	−0.251

**Table 2:** Minimum inhibitory concentration (MIC) and minimum fungicidal concentration (MFC) values (μg/mL) of the TdPR10.1 protein determined by the microdilution method.

Phytopathogens	CMI (µg/mL)	CMF (µg/mL)	CMF/CMI
*F. oxysporum*	>500	500	
*F. culmorum*	>500	-	
*F. graminearum*	500	500	1
*B. cinerea*	>500	500	
*A. niger*	500	500	1

## Data Availability

No datasets were generated or analyzed during the current study.

## Supplementary Materials

The following supporting information can be downloaded at: https://www.mdpi.com/article/10.3390/plants13223128/s1, Figure S1: (A) Alignment of the durum wheat PR10 proteins by using the muscle algorithm of MEGA 11 and visualized by genedoc software. (B) A specific Bet v 1 signature of TdPR10 created with the WebLogo tool. Figure S2. Identity analysis of TdPR10 proteins and other PR10 proteins from different species known by their functions: Pru av 1 from *Prunus avium* (sweet cherry) (O24248.1); Pyrc1 from *Pyrus communis* (AAC13315.1); Api g 1(P49372.1) from *Apium graveolens*; Dau c 1 (O04298.1); Cor a 1 (Q08407.3) from *Corylus avellana*; Ara h 8 from *Arachis hypogaea* (AAQ91847.1); Cas s 1 (ACJ23861.1) from *Castanea sativa*; *PmPr10-3.1* (AAL50006.1) from *Pinus monticola*; HbPR10 (XP_021658365.1) from *Hevea brasiliensis*; NbMLP28: MLP-like protein 28 from *Nicotiana benthamiana* (QEH62705.1); PBZ1: (BAA24277.1) from *Oryza sativa* Japonica; *Gly m 4l* (HQ913577.1) from *Glycine max*; Intracellular pathogenesis-related protein PinmIII from *Pinus monticola* (AAC33531.1) and Solyc09g090980 from *Solanum lycopersicum.* Figure S3: Production and purification of TdPR10-1 protein from *E. coli* strain. Coomassie blue staining of SDS PAGE gel. (MW), molecular weight markers; (Ex), Extract of TdPR10-1 protein after induction; (Pr), The final purified product of TdPR10-1 as obtained after thrombin cleavage. Table S1: List of primers used in QRT-PCR analysis.
