# Supplementary material for: Genome-Wide Identification of PR10 Family Members in Durum Wheat: Expression Profile and In Vitro Analyses of TdPR10.1 in Response to Various Stress Conditions
Source: Plants (Basel). 2024 Nov 7;13(22):3128. doi: 10.3390/plants13223128 (PMC11597350; doi:10.3390/plants13223128)
Supplement: Supplementary file 1 [file plants-13-03128-s001.zip › plants-3264158-supplementary.pptx]

## Slide 1
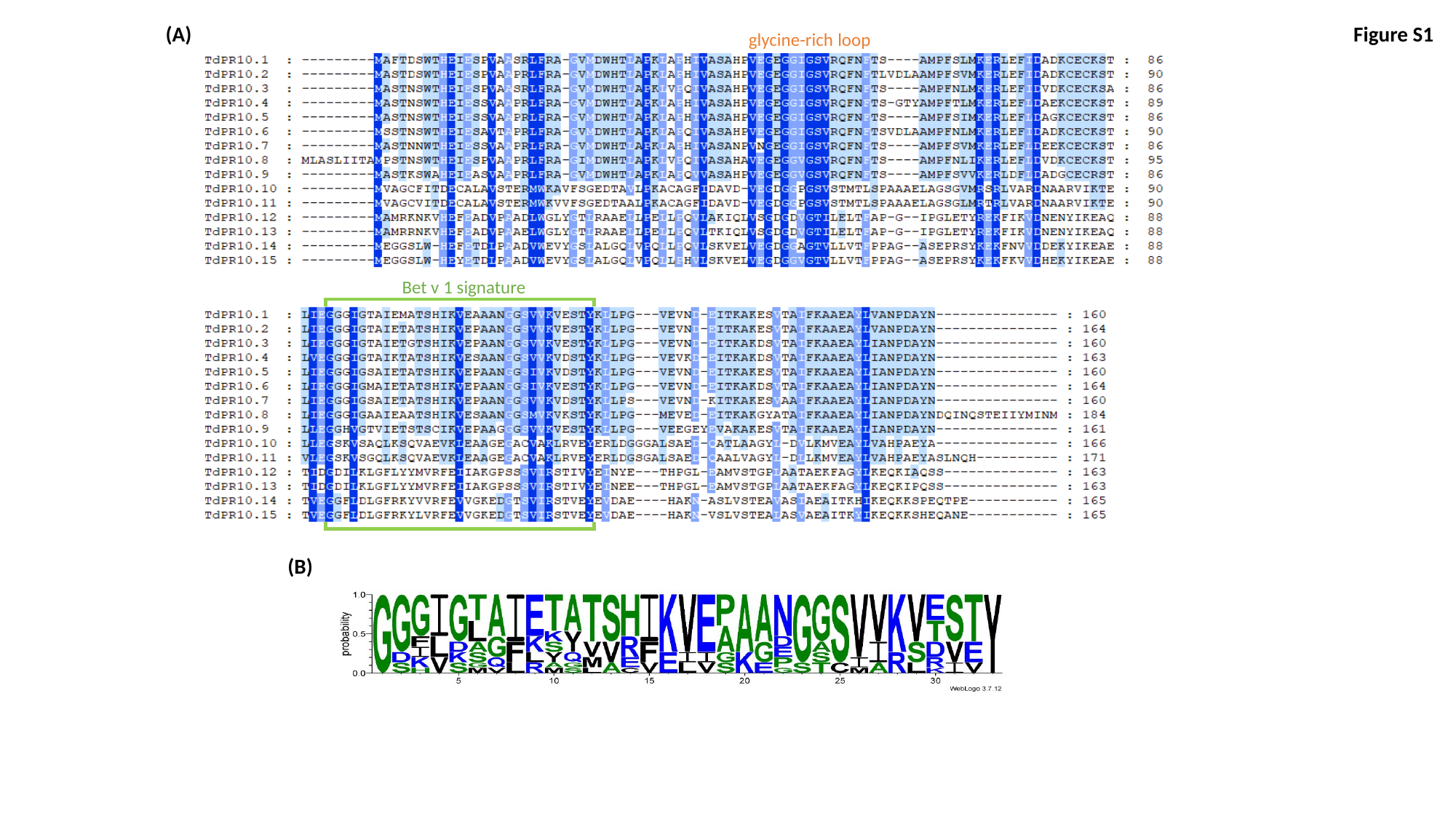

(A)
glycine-rich loop
Bet v 1 signature
(B)
Figure S1

## Slide 2
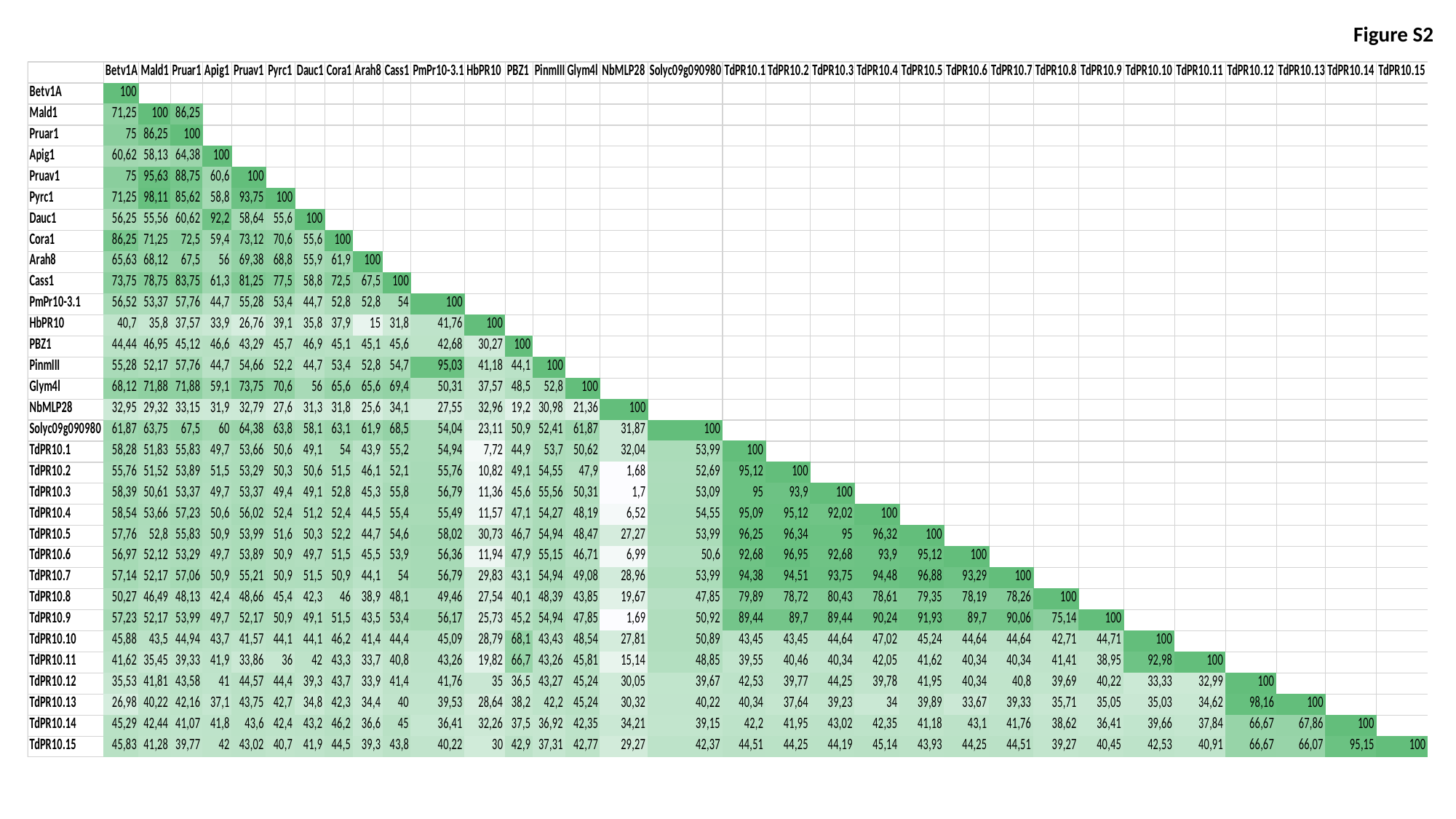

Figure S2

## Slide 3
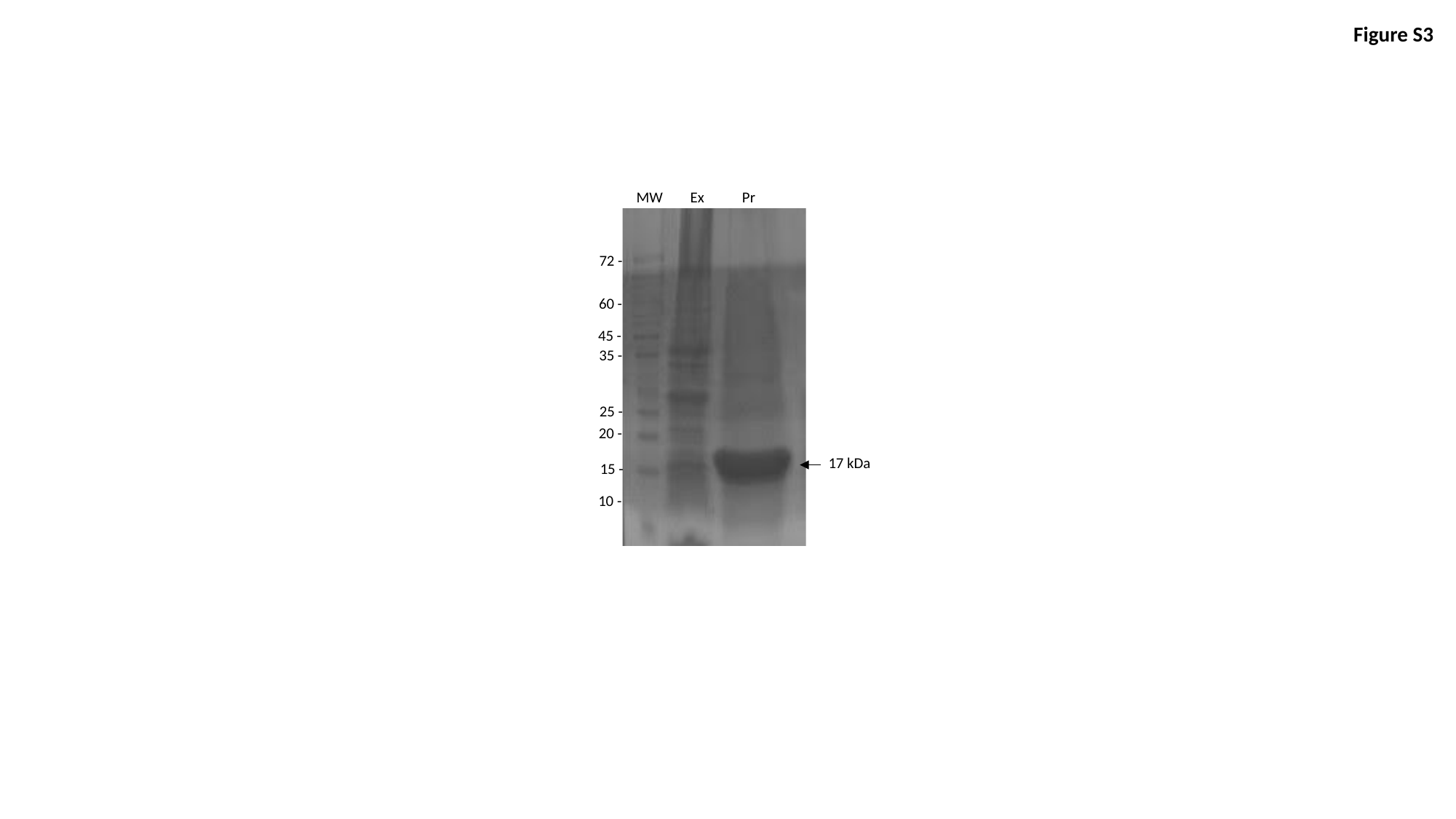

Figure S3
MW Ex Pr
72 -
60 -
45 -
35 -
25 -
20 -
17 kDa
15 -
10 -

## Slide 4
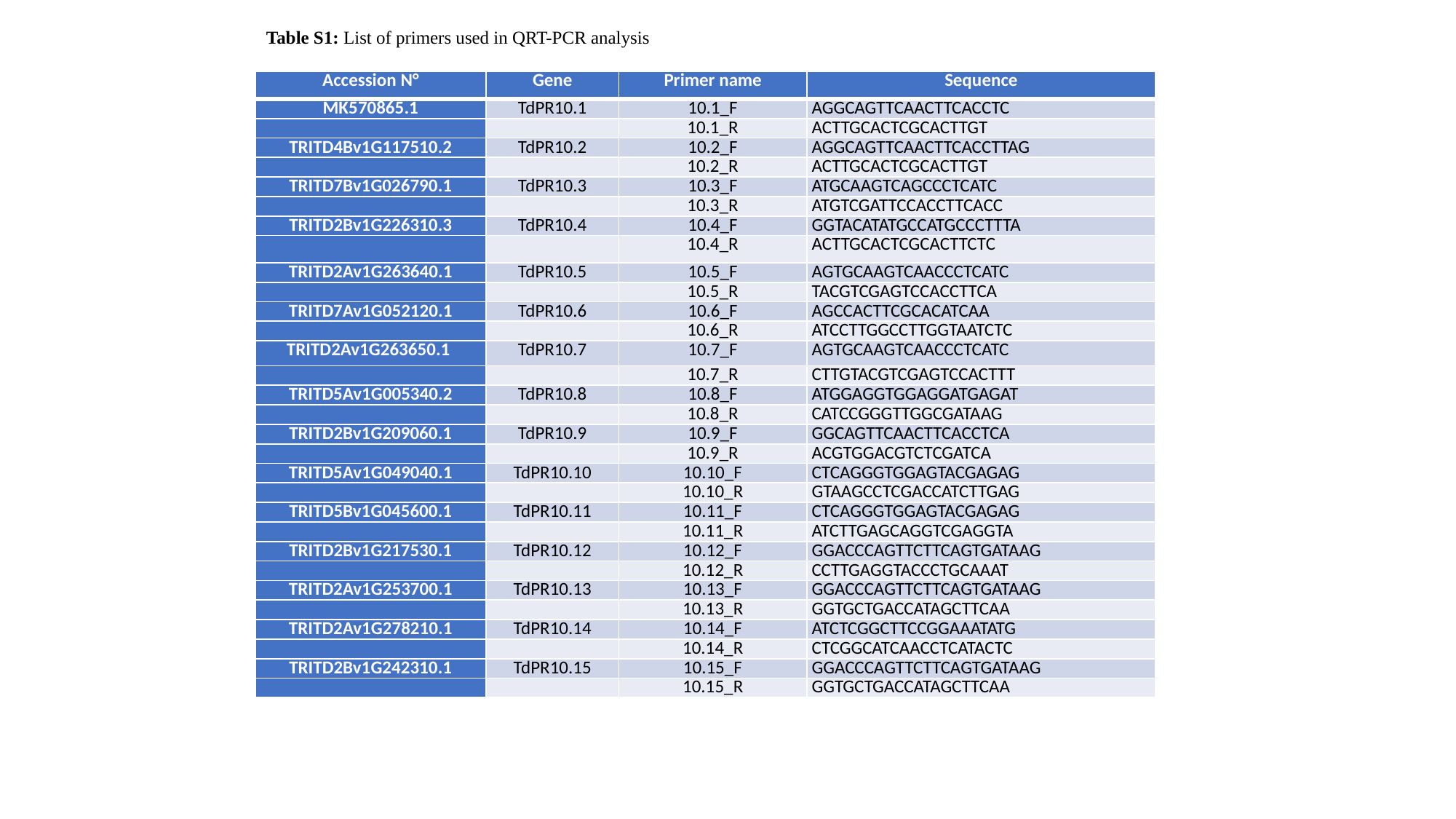

Table S1: List of primers used in QRT-PCR analysis
| Accession N° | Gene | Primer name | Sequence |
| --- | --- | --- | --- |
| MK570865.1 | TdPR10.1 | 10.1\_F | AGGCAGTTCAACTTCACCTC |
| | | 10.1\_R | ACTTGCACTCGCACTTGT |
| TRITD4Bv1G117510.2 | TdPR10.2 | 10.2\_F | AGGCAGTTCAACTTCACCTTAG |
| | | 10.2\_R | ACTTGCACTCGCACTTGT |
| TRITD7Bv1G026790.1 | TdPR10.3 | 10.3\_F | ATGCAAGTCAGCCCTCATC |
| | | 10.3\_R | ATGTCGATTCCACCTTCACC |
| TRITD2Bv1G226310.3 | TdPR10.4 | 10.4\_F | GGTACATATGCCATGCCCTTTA |
| | | 10.4\_R | ACTTGCACTCGCACTTCTC |
| TRITD2Av1G263640.1 | TdPR10.5 | 10.5\_F | AGTGCAAGTCAACCCTCATC |
| | | 10.5\_R | TACGTCGAGTCCACCTTCA |
| TRITD7Av1G052120.1 | TdPR10.6 | 10.6\_F | AGCCACTTCGCACATCAA |
| | | 10.6\_R | ATCCTTGGCCTTGGTAATCTC |
| TRITD2Av1G263650.1 | TdPR10.7 | 10.7\_F | AGTGCAAGTCAACCCTCATC |
| | | 10.7\_R | CTTGTACGTCGAGTCCACTTT |
| TRITD5Av1G005340.2 | TdPR10.8 | 10.8\_F | ATGGAGGTGGAGGATGAGAT |
| | | 10.8\_R | CATCCGGGTTGGCGATAAG |
| TRITD2Bv1G209060.1 | TdPR10.9 | 10.9\_F | GGCAGTTCAACTTCACCTCA |
| | | 10.9\_R | ACGTGGACGTCTCGATCA |
| TRITD5Av1G049040.1 | TdPR10.10 | 10.10\_F | CTCAGGGTGGAGTACGAGAG |
| | | 10.10\_R | GTAAGCCTCGACCATCTTGAG |
| TRITD5Bv1G045600.1 | TdPR10.11 | 10.11\_F | CTCAGGGTGGAGTACGAGAG |
| | | 10.11\_R | ATCTTGAGCAGGTCGAGGTA |
| TRITD2Bv1G217530.1 | TdPR10.12 | 10.12\_F | GGACCCAGTTCTTCAGTGATAAG |
| | | 10.12\_R | CCTTGAGGTACCCTGCAAAT |
| TRITD2Av1G253700.1 | TdPR10.13 | 10.13\_F | GGACCCAGTTCTTCAGTGATAAG |
| | | 10.13\_R | GGTGCTGACCATAGCTTCAA |
| TRITD2Av1G278210.1 | TdPR10.14 | 10.14\_F | ATCTCGGCTTCCGGAAATATG |
| | | 10.14\_R | CTCGGCATCAACCTCATACTC |
| TRITD2Bv1G242310.1 | TdPR10.15 | 10.15\_F | GGACCCAGTTCTTCAGTGATAAG |
| | | 10.15\_R | GGTGCTGACCATAGCTTCAA |
